# Allopurinol Prevents the Lipogenic Response Induced by an Acute Oral Fructose Challenge in Short-Term Fructose Fed Rats

**DOI:** 10.3390/biom9100601

**Published:** 2019-10-11

**Authors:** Fernando E. García-Arroyo, Fabiola Monroy-Sánchez, Itzel Muñoz-Jiménez, Guillermo Gonzaga, Ana Andrés-Hernando, Cecilia Zazueta, J. Gabriel Juárez-Rojas, Miguel A. Lanaspa, Richard J. Johnson, L. Gabriela Sánchez-Lozada

**Affiliations:** 1 Department of Cardio-Renal Physiopathology, INC Ignacio Chávez, Mexico City 14080, Mexico; jonibertojr@hotmail.com (F.E.G.-A.); fabyms@gmail.com (F.M.-S.); itzel.morrison@icloud.com (I.M.-J.); ggonzaga49@gmail.com (G.G.); 2 Renal Diseases and Hypertension University of Colorado, Aurora, CO 80045, USA; Ana.AndresHernando@cuanschutz.edu (A.A.-H.); Richard.Johnson@cuanschutz.edu (R.J.J.); 3 Department of Cardiovascular Biomedicine, INC Ignacio Chávez, Mexico City 14080, Mexico; czazuetam@hotmail.com; 4 Department of Endocrinology, INC Ignacio Chávez, Mexico City 14080, Mexico; gaboyk2@gmail.com

**Keywords:** hepatic steatosis, mitochondria, mitochondrial complex 1

## Abstract

We investigated whether short term high fructose intake may induce early hepatic dysfunction in rats and to test whether allopurinol treatment may have beneficial effects. Twenty male Sprague-Dawley rats received 20% fructose in drinking water (10 treated with allopurinol and 10 received vehicle) and 10 control rats received tap water. After 14 days, the hepatic response to an acute fructose load was evaluated, and in fasted animals, respirometry studies in freshly isolated mitochondria were performed. In fasting rats, we did not find differences in systemic or hepatic uric acid and triglyceride concentrations among the groups, but mitochondrial respiratory control rate was significantly decreased by high fructose feeding and correlated with a reduced expression of Complex I, as well as decreased aconitase-2 activity. On the other hand, in fructose fed rats, an acute fructose load increased systemic and hepatic uric acid, triglycerides and oxidative stress. Fructose feeding was also associated with fructokinase and xanthine oxidase overexpression and increased liver de novo lipogenesis program (fatty acid synthase (FAS) and cell death-inducing DFFA-like effector C (CIDEC) overexpression, ATP citrate lyase (ACL) and acetyl coA carboxylase (ACC) overactivity and decreased AMP-activated protein kinase (AMPk) and endothelial nitric oxide synthase (eNOS) activation). Allopurinol treatment prevented hepatic and systemic alterations. These data suggest that early treatment with xanthine oxidase inhibitors might provide a therapeutic advantage by delaying or even halting the progression of non-alcoholic fatty liver disease (NAFLD).

## 1. Introduction

The role of high fructose intake (HFI) is well recognized in the development of lipids abnormalities and metabolic syndrome in laboratory animals and in humans [1]. A well-described effect of HFI is the relative depletion of adenosine triphosphate (ATP), resulting from the synthesis of the first byproduct of its metabolism, fructose 1-phosphate, which is mediated by the enzyme ketohexokinase (KHK) in the liver and other organs [2,3,4]. Contrary to glucokinase, KHK does not have a negative feedback mechanism, it has high affinity for fructose, and its protein expression is increased in response to HFI [5,6]. All of these characteristics explain why intrahepatic ATP and phosphate levels plummet after a continuous and abundant fructose intake and tend to be low in long-term fructose exposure [7]. A direct consequence of ATP and phosphate depletion is the activation of adenosine monophosphate (AMP) deaminase, which starts the pathway of purines degradation leading to the increased synthesis of uric acid [2]. Our group reported that uric acid might not be just an inert byproduct of fructose metabolism, but instead, it seems to have an active participation in the hepatic and metabolic alterations mediated by fructose. Hence, it was found that xanthine oxidase inhibition, with allopurinol or febuxostat and the uricosuric benzodiarone were able to improve the metabolic syndrome induced by fructose [8,9]. In a pilot study in overweight humans, we reported that allopurinol treatment concomitant to 200 g/day of fructose for two weeks prevented the increase in newly diagnosed metabolic syndrome observed in subjects that not received allopurinol [10]. In addition, in human hepatoma derived hepatocytes (HepG2 cells) the intracellular lipid accumulation mediated by fructose incubation was prevented by coincubation with allopurinol [11,12]. The role of uric acid on hepatic steatosis was further demonstrated in HepG2, in which exposure to uric acid upregulated the expression of KHK and fatty acid synthase through mechanisms associated with increased oxidative stress and the translocation of carbohydrate response element binding protein (ChREBP) into the nuclei [11,12]. In addition, in experimental hyperuricemia in rodents, hepatic steatosis was mediated by the activation of NACHT, LRR and PYD domains-containing protein 3 (NLRP3) inflammasome and the polyol pathway with the subsequent endogenous fructose synthesis [13,14] therefore inducing a vicious circle. 

Hepatic steatosis is the earliest manifestation of metabolic syndrome in the liver, and it is followed by the development of non-alcoholic steatohepatitis [15] In addition, high fructose intake has been associated with the development of fatty liver through the promotion of de novo lipogenesis and intrahepatic lipid accumulation, inhibition of long-chain fatty acids mitochondrial β-oxidation, and triglycerides formation [16]. Despite these findings, animal models have shown that fatty liver development takes at least 8 weeks in rodents exposed to HFI [17], and liquid fructose has been found to induce metabolic alterations faster in comparison to fructose contained in solid food [18,19]. Thus, the present study aimed to investigate whether short term HFI may induce hepatic abnormalities and to test whether allopurinol treatment could have a beneficial effect on such putative early hepatic alterations induced by HFI in rats.

## 2. Materials and Methods

All the studies were approved by the Internal Animal Care and Use Committee (Permit No INC/CICUAL/PIL/002/2019) and were conducted in agreement with the National Institutes of Health guide for the care and use of laboratory animals (NIH Publications No. 8023, revised 1978). We studied male Sprague-Dawley rats obtained from the Instituto Nacional de Cardiología Ignacio Chávez vivarium. Rats (360–400 g, 14–15 weeks old, virgin rats) were allocated in individual acrylic cages in the following groups: 10 rats received 20% fructose in drinking water plus vehicle (water) by gavage (F20% group), 10 rats received 20% fructose in drinking water plus allopurinol (5 mg/day in 1 mL of water) by gavage (F20% + AP group), and 10 rats received tap water and were included as controls. All groups were followed for 14 days. The dose of fructose was chosen as it has been reported that the highest consumers of added sugars in the USA had a fructose intake of 20–25% of caloric intake [20]. At the end of the follow-up, urine was collected in metabolic cages during 16 h with food and drinking fluid ad libitum. Half of the animals in each group (*n* = 5) were used to evaluate the response of an acute fructose load by the liver (2 g/kg/BW). To test the effects of high glucose intake and its liver handling after and acute load 4 rats were given glucose 20% for 14 days and received an acute load of glucose (2 g/kg/BW). In the remaining fasted animals (*n* = 5 each group), liver tissue was excised to perform respirometry studies in freshly isolated mitochondria and to measure the intrahepatic and plasma concentrations of uric acid and triglycerides.

### 2.1. Effect of an Acute Fructose/Glucose Load in the Liver

Rats that received 20% fructose or 20% glucose for 14 days were fasted for 16–18 h and then received an acute oral load of fructose or glucose (2 g/kg BW). After 2.5 h, animals were anesthetized with isoflurane, exsanguinated from the abdominal aorta and liver right lobe collected. Samples of plasma were frozen at −20 °C, and liver samples were frozen in liquid nitrogen and stored at −80 ºC until processing.

### 2.2. Plasma and Liver Uric Acid 

Plasma and liver uric acid (Sekisui Diagnostics, San Diego, CA, USA), triglycerides (Sekisui Diagnostics, San Diego, CA, USA) were measured in samples taken in fasting and after the acute o fructose load. Also, in liver samples from fasted or acute fructose/glucose loaded rats, markers of oxidative stress (lipid peroxidation and protein oxidation) and adenosine diphosphate/adenosine triphosphate (ADP/ATP) ratio (Sigma Aldrich, Darmstadt, Germany) were evaluated.

### 2.3. Mitochondria Isolation

Mitochondria were isolated from the renal cortex by differential centrifugation as previously described [21]. Proteins were measured by the Bradford method.

#### 2.3.1. Mitochondrial Oxygen Consumption

Mitochondrial oxygen consumption was measured using a Clark-type oxygen electrode (Yellow Springs Instruments, Yellow Springs, OH, USA). State 4 respiration rate was evaluated in 1.5mL of basic medium containing 125 mM KCl (Sigma-Aldrich, St. Louis, MO, USA), 10mM HEPES, 3 mM Pi and 10 mM succinate plus 1 µg/mL rotenone (Sigma-Aldrich, St. Louis, MO, USA), or 5 mM sodium glutamate (Sigma-Aldrich, St. Louis, MO, USA) plus 5 mM sodium malate (Sigma-Aldrich, St. Louis, MO, USA). State 3 respiration rate was measured after the addition of 200 µM ADP (Sigma-Aldrich, St. Louis, MO, USA). The respiratory control index (RC) was calculated as the ratio between state 3/state 4 rates. Uncoupled respiration was measured by adding CCCP (Sigma-Aldrich, St. Louis, MO, USA); phosphorylation efficiency was calculated from the added amount of ADP and the total amount of oxygen consumed during state 3 (ADP/O ratio) [22].

#### 2.3.2. Aconitase Activity

Aconitase activity was measured in isolated mitochondria as the formation of cis-aconitate from isocitrate at 240 nm in Tris-HCl buffer, pH 7.4 in a medium containing isocitrate (Sigma-Aldrich, St. Louis, MO, USA) and MnCl_2_ (Sigma-Aldrich, St. Louis, MO, USA). One unit was defined as the amount of enzyme necessary to produce 1 µmol of cis-aconitate/min [23].

### 2.4. Protein Extraction and Immunoblotting

Soluble fraction of hepatic tissue was extracted for 30 min on ice bath in MAP kinase buffer [in mmol/l 25 HEPES (pH 7.4 Sigma-Aldrich, St. Louis, MO, USA), 150 NaCl (Sigma-Aldrich, St. Louis, MO, USA), 4 EDTA(Sigma-Aldrich, St. Louis, MO, USA), 25 NaF (Sigma-Aldrich, St. Louis, MO, USA), and 1 Na_3_VO4 (Sigma-Aldrich, St. Louis, MO, USA) with 1% (*v*/*v*) NP-40 (Sigma-Aldrich, St. Louis, MO, USA) with Complete Protease Inhibitor and Phosphatase inhibitor (Roche Diagnostics, Indianapolis, IN, USA)] and protein content was determined by the Bradford method. Thirty micrograms of protein were loaded per lane for SDS/PAGE (10% *w*/*v*) separation and then transferred to PVDF membranes (Merck Millipore Ltd, Cork, Ireland). Membranes were incubated overnight at 4 °C, with the following primary antibodies: OXPHOS (Abcam, ab110413, 1:2500 dilution, Cambridge, UK), Aconitase-2 (Genetex, GTX114233, 1:2000 dilution, Irvine, CA, USA) Bax (Santacruz Biotechnology, sc-7480, 1:1500 dilution, Santacruz, CA, USA) Bcl2 (Santacruz Biotechnology, sc-509, 1:1000 dilution. Santacruz, CA, USA) ATP citrate lyase (Genetex, GTX112387, 1:3000 dilution. Irvine, CA, USA) phospho T447 + S451 ATP citrate lyase (Abcam, ab53007, 1:3000 dilution. Cambridge, UK), Acetyl CoA carboxilase (Genetex, GTX132081, 1:2500 dilution. Irvine, CA, USA), Fatty acid synthase (Genetex, GTX109833, 1:5000 dilution. Irvine, CA, USA) KHK (Genetex, GTX109591, 1:10,000. Irvine, CA, USA) Xanthine oxidase (Santacruz Biotechnology, sc-398548, 1:1000 dilution. Santacruz, CA, USA) CIDEC (Abnova, H00063924, 1:2000 dilution. Jhongli, Taiwan) SREBP1 (Genetex, GTX79299, 1:3000 dilution. Irvine, CA, USA), PCNA (Genetex, GTX100539, 1:2500 dilution. Irvine, CA, USA) AMPKα (Cell Signaling, 2532, 1:3000 dilution. Danvers, MA, USA) phosphor AMPKα (Cell Signaling, D4D6D),1:2000 dilution. Danvers, MA, USA) eNOS (Genetex, GTX129843, 1:4000 dilution. Irvine, CA, USA) phosphor Ser1177 eNOS (Genetex, GTX129058, 1:2000 dilution. Irvine, CA, USA) and visualized by using a horseradish peroxidase (HRP) secondary antibody (Cell Signaling, 7074, Danvers, MA, USA) and the ECL Clarity (Bio-Rad, Hercules, CA, USA). Immunoblots were analyzed with Image Studio Lite 5.2 software (Licor Biosciences, Lincoln, NE, USA).

### 2.5. Statistical Analysis

The data were analyzed using Prism 8 (GraphPad Software, San Diego CA, USA). Results are presented as mean ± SD and were analyzed by one-way ANOVA. The significance was set at *p* < 0.05. Post hoc analysis was performed using the Bonferroni test.

## 3. Results

### 3.1. General Parameters and Renal Function

Water intake was 35 ± 2.8 mL/d in normal rats. Fructose in drinking water increased significantly the mean water intake to 58 ± 5 mL/d in F20% and 53 ± 10 mL/d in F20% + AP groups (Table 1). In both fructose groups, food (chow) intake decreased compared to control rats (Table 1), indicating compensation for increased calories ingested in water. There were no changes in body weight between control and F20% at the end of the study. However, F20% + AP was associated with BW loss (Table 1). Fasting plasma and intrahepatic TG concentrations were not different among the groups and fasting uric acid intrahepatic concentration was lower in allopurinol treated animals while plasma uric acid was not different among the groups (Table 1). As allopurinol may be associated with the formation of renal xanthine calculus in rats, we quantified urinary protein excretion and calculated creatinine clearance in order to evaluate kidney function. As shown in Table 1, proteinuria and CrCl were similar among the groups at the end of the study. Therefore, the dose of allopurinol administrated was well tolerated by the animals.

### 3.2. Allopurinol Prevented Acute ATP Depletion

**Figure 1:**
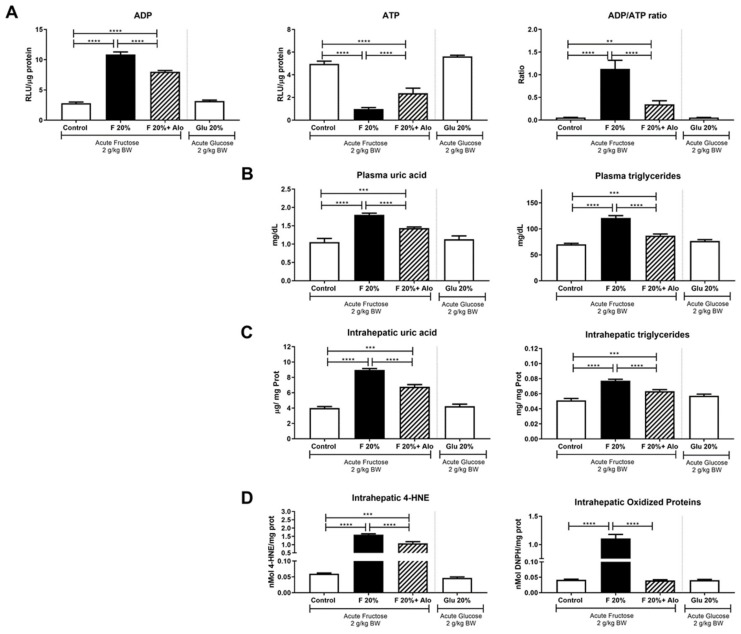
Effect of an acute load of fructose in short-term high fructose fed fasted rats. (**A**) An acute load of fructose (2 g/kg BW) induced a significant increase in hepatic ADP in parallel with ATP depletion, thus increasing ADP/ATP ratio. Allopurinol treatment prevented such effect. (**B**) Plasma and (**C**) hepatic uric acid and triglycerides were increased by fructose acute load and allopurinol treatment prevented this effect. (**D**) Fructose induced hepatic oxidative stress that was prevented by allopurinol. On the other hand, glucose acute load did not induce any of the deleterious effects exerted by fructose in short term high glucose fed rats (white bars in all graphs). ** = *p* < 0.01; *** = *p* < 0.001; **** = *p* < 0.0001.

As there were no changes in systemic neither in hepatic uric acid and triglycerides concentrations after 14 days of increased fructose intake, we evaluated the effect of an acute oral dose of fructose in fasted rats. After 2.5 h of fructose load, we observed a sharp increment in ADP/ATP ratio caused by an ATP decrement and ADP increment in the liver of rats that received 20% fructose beverage during 2 weeks (Figure 1A). ATP depletion caused an increase in plasma and liver uric acid and triglyceride concentrations in the group that received 20% fructose (Figure 1B,C). Hepatic lipid peroxidation and protein oxidation were also increased in fructose rats after acute exposure to fructose (Figure 1D). Allopurinol dosed with fructose prevented these hepatic effects. In addition, exposure to an acute glucose load did not produce any deleterious effect in rats that received 20% glucose for two weeks (Figure 1A–D).

### 3.3. Allopurinol Prevented Hepatic Mitochondria Uncoupling

**Figure 2:**
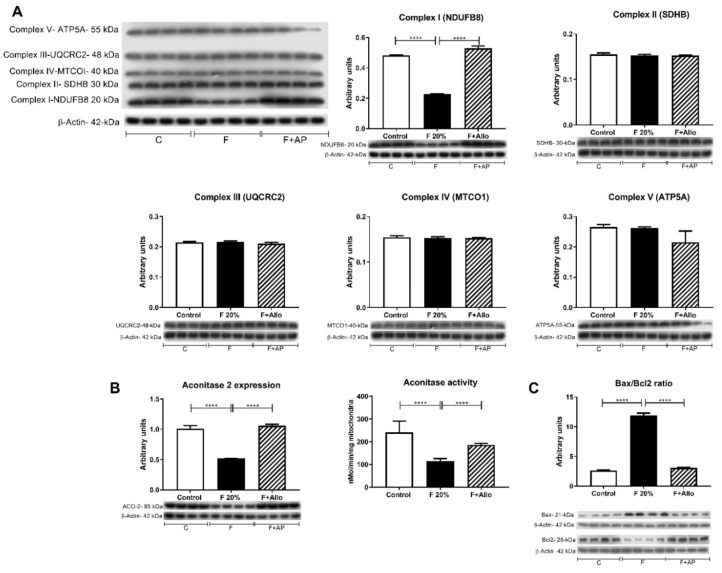
Mitochondrial complexes expression by western blot, aconitase 2 expression and activity and Bax/Bcl2 ratio. (**A**) Short term high fructose feeding only decreased the expressions of mitochondrial respiration Complex 1 and (**B**) aconitase 2. Fructose also decreased aconitase 2 activity (**B**). On the contrary, Bax/Bcl2 ratio was increased by high fructose feeding (**C**). Allopurinol treatment prevented all these effects. For western blotting, 4 out 5 randomly selected samples per group were analyzed. **** = *p* < 0.0001.

To study whether there were mitochondrial changes induced by fructose intake, we evaluated the whole respiratory chain in isolated mitochondria of fasted rats. Oxygen consumption was stimulated by feeding Complex 1 with malate/glutamate and Complex 2 with succinate/rotenone (Table 2). Significant elevation in State 4 respiration was observed in rats given fructose (F20%), whereas, State 3 (coupled to ATP synthesis) was similar to those of control animals, resulting in a significant decrease of the Respiratory Control rate in the F20% group (Table 2). The maximal respiratory rate, measured by the addition of the uncoupling agent CCCP (to dissipate chemiosmotic gradient), was not different in F20% treated rats compared to normal rats. Allopurinol treatment (F20% + AP) prevented the changes in malate/glutamate RCR, and State 4. Thus, the values recorded for these parameters were similar with the control group. In succinate/rotenone stimulated respiration, no changes were detected in state 4, state 3 and RCR among the groups (Table 2). Maximal respiration rate, measured by the addition of the uncoupling agent CCCP, was found to be lower in F20% + AP group compared to control and F20%. In addition, we evaluated the expression of the five mitochondrial respiration complexes. We only found a significantly decreased expression in Complex 1 in 20% fructose rats, that was prevented by allopurinol (Figure 2A).

### 3.4. Allopurinol Prevented Apoptosis

As Bcl2 and Bax proteins play important roles in regulating mitochondrial function and its ratio is associated with caspase-independent apoptosis, we evaluated their expression. A twenty percent fructose beverage increased the Bax/Bcl2 ratio, and allopurinol precluded this effect (Figure 2C).

### 3.5. Immunoblotting for KHK and XO

**Figure 3:**
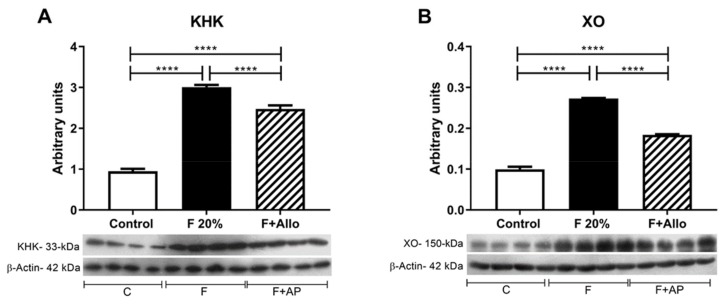
Fructokinase (KHK) and XO (xanthine oxidase) protein expression in liver. High fructose intake induced the upregulation of KHK (**A**) and XO (**B**) expression in liver. Allopurinol prevented this effect. For western blotting, 4 out of 5 randomly selected samples per group were analyzed. ****= *p* < 0.0001.

Fructose intake has been associated with the overexpression of KHK (30). In this study, we tested whether fructose intake can also upregulate XO expression. Fructose induced the overexpression of both enzymes; allopurinol concomitant treatment partially prevented this effect (Figure 3A,B).

### 3.6. Immunoblotting for Markers of Lipid Metabolism after an Acute Load of Fructose

**Figure 4:**
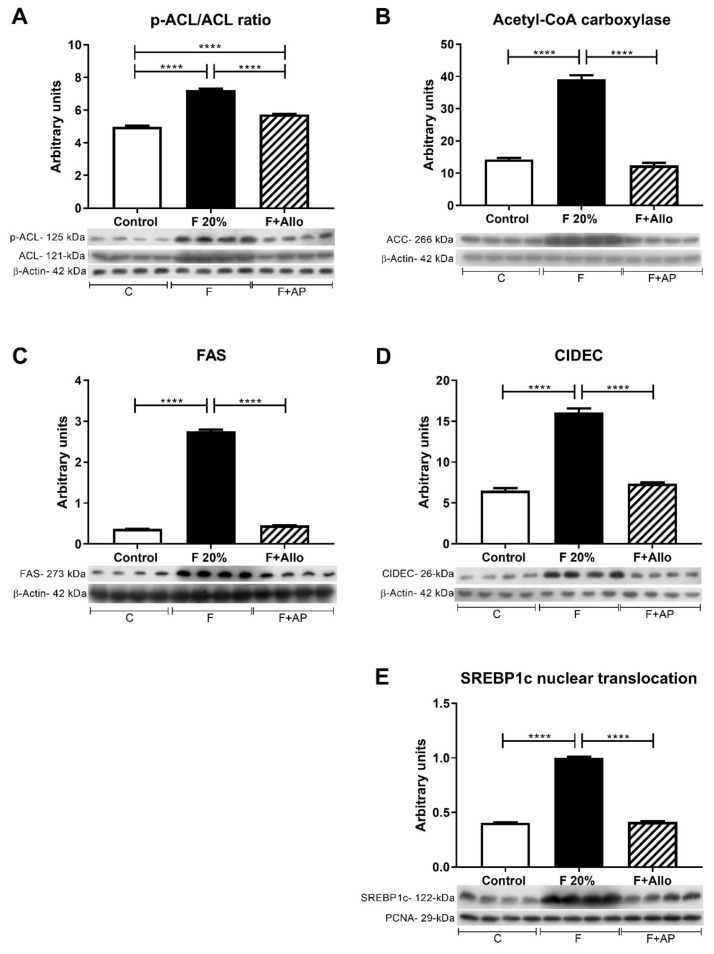
Markers of lipid metabolism after an acute load of fructose. In high fructose fed rats challenged with and acute load of fructose, there was an increased activation of ATP citrate lyase (**A**) and acetyl CoA carboxylase (**B**) as well as an increased expression of fatty acid synthase (**C**) and CIDEC (**D**). These effects were in parallel with an increased nuclear translocation of the transcription factor SREBP1c (**E**). Allopurinol treatment prevented all these effects. For western blotting, 4 out of 5 randomly selected samples per group were analyzed. **** = *p* < 0.0001.

To assess the lipogenic response to an acute load of oral fructose, we evaluated the following enzymes and proteins implicated in the de novo hepatic lipogenesis p-ATP citrate lyase/ATP citrate lyase ratio (p-ACL/ACL acetyl CoA carboxylase (ACC), fatty acid synthase (FAS), cell death-inducing dFF45-like effector C (CIDEC) and the nuclear translocation of the sterol regulatory element-binding protein 1 (SREBP1c) (Figure 4). Fructose significantly increased the level of activation of ACL compared to control rats, showed as a rise in the p-ACL/ACL ratio. Allopurinol treatment of fructose-fed rats prevented the activation of ACL induced by fructose (Figure 4A). Fructose also increased ACC expression, and allopurinol prevented this effect (Figure 4B). Likewise, FAS and CIDEC were upregulated by high fructose intake, and allopurinol dosing prevented this upregulation (Figure 4C,D). These effects occurred in parallel with a significant increment in SREBP1c nuclear translocation induced by high fructose intake and were prevented by allopurinol (Figure 4E)

### 3.7. Immunobloting for p-AMPk/AMPk Ratio and p-eNOS/eNOS Ratio

**Figure 5:**
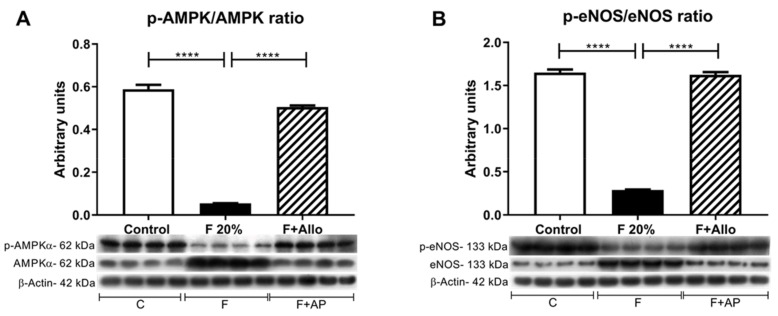
AMPk and eNOS activation is reduced after an acute load of fructose. In high fructose fed rats challenged with an acute load of fructose, there was a decreased activation of AMPk (**A**) and eNOS (**B**). Allopurinol treatment prevented all these effects. For western blotting, 4 out 5 randomly selected samples per group were analyzed. **** = *p* < 0.0001.

Activation of the AMPk has been demonstrated to attenuate liver steatosis, therefore we evaluated the effect of allopurinol in this parameter. High fructose feeding reduced the activation of AMPk, noted by a reduction in phospho (p)AMPk/AMPk ratio compared to control. Allopurinol rescued the activation of AMPk (Figure 5A). Fructose intake is also associated with endothelial dysfunction. In the liver, such a defect can induce hepatic insulin resistance. Even though fructose feeding induced a marked upregulation of eNOS protein, fructose reduced its phosphorylation compared to control (lower peNOS/eNOS ratio). Allopurinol treatment rescued the phosphorylation of eNOS in fructose-fed rats (Figure 5B).

## 4. Discussion

High fructose diets, especially when the fructose is given in liquids, are well-known to cause obesity and metabolic syndrome, but typically it takes several months. Indeed, short-term feeding of fructose (< 4 weeks) is often associated with minimal metabolic findings, leading some investigators to propose that fructose is benign [24]. However, there is some evidence that if subjects have preexisting insulin resistance, that they may be more sensitive to the effects of fructose [25].

One potential explanation is the fact that normally the gut has low expression of Glut5, the fructose transporter, and with exposure to fructose, these transporters can be induced [26,27]. Fructose (or sucrose) also increases the expression of fructokinase in the intestine and liver [6]. Indeed, there is some evidence that children with obesity and fatty liver show enhanced fructose absorption and metabolism, that could be consistent with higher past exposure to sugars [28].

Here, we evaluated the effect of fructose on metabolic parameters in rats that had been ‘primed’ with two weeks of fructose provided in the water. During these two weeks, the fructose fed fats showed no difference in a variety of metabolic parameters (weight, fatty liver, serum lipids, etc.) compared to control rats. Nevertheless, when rats were exposed to an acute load of fructose, they showed dramatic changes in metabolism driven by alterations in ATP and mitochondrial function, and of interest, most of these mechanisms were mediated in part by the generation of uric acid in the nucleotide degradation pathway, as shown by the effects of allopurinol.

Fructose given as a 20% solution in drinking water did not induce an increment in body weight in this short-term study (14 days) compared to control rats receiving tap water. Nevertheless, allopurinol treatment induced a mild but significant weight loss despite equivalent food and water intake compared to F20% of animals. Allopurinol has been rarely associated with acute liver injury, mainly in older patients with concomitant kidney damage, as kidney is more sensitive to allopurinol toxic effects. Thus, we believe that the weight loss observed in F20%+Allopurinol rats was not related to renal toxicity by allopurinol since proteinuria and creatinine clearance were comparable among the studied groups, suggesting that allopurinol was well tolerated. Thus, the effect of allopurinol might be related to lower feed efficiency, e.g., less weight gain per calorie ingested. Such an allopurinol effect on body weight has also been observed in overweight humans on a high fructose diet [29].

Previous studies have shown that the hepatic alterations induced by fructose may take eight weeks or longer to develop in rodents depending on the dose [30]. Therefore, it was expected that after 14 days of fructose exposure, there were no hyperuricemia either hypertriglyceridemia in the fasting state. Since fructose metabolism is relatively fast after an oral load [4], we studied the effect of an acute load of fructose in this short-term intake of high fructose in fasted rats. Two hours and a half after the oral load, we observed a significant increment in ADP/ATP ratio as well as in plasma and intrahepatic uric acid and triglycerides; the markers of lipid peroxidation and protein oxidation were also significantly increased in liver. Interestingly, an equimolar acute load of glucose in short term high-glucose intake rats did not produce such deleterious effects. In addition, allopurinol given alongside with fructose prevented the systemic and hepatic harmful effects induced by the fructose load. The therapeutic benefit conferred by allopurinol was likely mediated by the prevention of KHK overexpression induced by fructose and uric acid as has been previously reported [12,14]. Xanthine oxidase is overexpressed in high fat diet induced-fatty liver [31], and these studies showed that also fructose can up-regulate its expression. As allopurinol prevented xanthine oxidase overexpression, this effect likely provided additional hepatoprotection. Thus, these studies showed that short term high fructose intake induces early hepatic alterations and confirms that allopurinol provides therapeutic benefit to prevent hepatic steatosis.

Since hepatic mitochondrial dysfunction is one of the earliest manifestations of liver steatosis, fructose as well as uric acid is associated with mitochondrial oxidative stress [11], we measured the respiratory capacity in isolated mitochondria of fasted animals. Respiratory control rate (tightness of the coupling between respiration and phosphorylation) using malate-glutamate as substrates, was lower in fructose-fed animals compared to the control group. This effect resulted from an increased state 4 in fructose animals with no changes in state 3. Allopurinol treatment fully prevented this effect and increased ADP/O2, suggesting that this treatment improved the mitochondrial coupling. Maximal oxygen consumption induced by CCCP, was reduced in the F20% + allopurinol as compared with N and F20%, this finding was unexpected as RCR values were not affected by the allopurinol treatment. As no overall bioenergetic dysfunction was observed in F20%-Allo rats, this effect is likely not related to mitochondrial function. In addition, preservation of State 4 indicates reduced proton leakiness. On the other, hand, when the respiratory chain was fed with succinate-rotenone, no changes in state 3, state 4 and RCR were observed among the groups. By western blot, we observed that complex 1 expression was significantly reduced in fructose-fed rats. As mitochondrial complex 1 is profoundly affected by oxidative stress, we also evaluated aconitase-2 activity as this enzyme is susceptible to oxidative stress. We found decreased aconitase-2 activity and expression in high fructose-fed rats, and this effect was prevented by allopurinol. These results suggest that augmented oxidative stress, partially mediated by increased synthesis of uric acid-induced by high fructose intake [11] results in mitochondrial dysfunction. Interestingly, it has been shown that rotenone (Complex 1 inhibitor) partially prevented the endoplasmic reticulum stress and steatosis induced by uric acid in HepG2 cells [32].

Previously it was shown that high fructose diet induces apoptosis of the hepatic tissue [33], we confirmed such results as Bax/Bcl2 ratio was increased in high fructose-fed rats. Allopurinol treatment was also useful to prevent this effect.

One of the prolipogenic effects of fructose is related to its ability to stimulate the activity and expression of the enzymes associated with fatty acid synthesis [30]. In this regard, inhibition of aconitase-2, accomplished by both fructose and uric acid-induced oxidative stress and increased cytoplasmic citrate [11] being this the first step that activates fatty acid synthesis by stimulating ATP citrate lyase. Oxidative stress also activates SREBP-1c, which downstream induces lipogenesis by increasing acetyl CoA carboxylase activation and fatty acid synthase overexpression [32,34]. In the present studies, we observed such effects mediated by short term high fructose intake, and allopurinol treatment prevented the lipogenic response induced by an acute fructose load. In addition, SRBP1c also enhanced the expression of the cell death-inducing DFFA C (DNA fragmentation factor-α)-like effector C (CIDEC) which plays an important role controlling the formation of unilocular lipid droplets for triglyceride intracellular storage in adipocytes and its upregulation has been reported in fatty liver as well [35,36]. The results of the present studies support that notion and high fructose intake induced the upregulation of CIDEC while allopurinol cotreatment prevented this effect. CIDEC has also been found that promotes apoptosis when it is expressed outside the lipid droplet context by a mechanism that includes caspase 9, −7 and −3 cleavage and the release of mitochondrial cytochrome C [37]. In the present studies, we observed a parallel increment in both CIDEC and Bax/Bcl2 ratio, suggesting that apoptosis of hepatic cells may be, at least partially mediated, by the increase in CIDEC expression in high fructose-fed rats. As allopurinol prevented CIDEC overexpression, concurrently avoided the increase in apoptosis.

Additional protective effects of allopurinol were that maintained the activation state of AMPk and eNOS, as inactivation of both enzymes has been associated with liver steatosis [38,39]. In contrast, short term fructose significantly reduced the activation of both enzymes. In this regard, it has been shown that CIDEC upregulation significantly reduced AMPk activity [40].

## 5. Conclusions

In summary, short-term fructose exposure induces early manifestations of systemic and hepatic alterations only noticed when an acute fructose load is administered and ratify the importance of increased uric acid synthesis secondary to fructose metabolism by fructokinase in these deleterious effects. These data also suggest that an early treatment with xanthine oxidase inhibitors might provide therapeutic advantage by delaying or even halting the progression of non-alcoholic fatty liver disease.

## Figures and Tables

**Table 1:** General parameters.

	Control	F 20%	F 20% + AP
Mean food intake, g/d	18 ± 1	14 ± 2 *	14 ± 1 *
Mean fluid intake, mL/d	35 ± 3	58 ± 5 *	53 ± 10 *
Delta Body weight, g	−6 ± 7	8 ± 8	−17 ± 19 °
Fasting plasma uric acid (mg/dL)	1 ± 0.28	0.73 ± 0.21	0.89 ± 0.28
Fasting plasma TG (mg/dL)	80 ± 18	83 ± 25	74 ± 34
Fasting intrahepatic uric acid (µg UA/mg prot)	5.3 ± 1	4.8 ± 1.1	2.5 ±0.2 *°
Fasting intrahepatic TG (mg TG/mg prot)	0.06 ± 0.01	0.04 ± 0.009	0.04 ± 0.006
Uprot, mg/16 h	14 ± 3	13 ± 2	16 ± 6
CrCl, mL/min	1.26 ± 0.2	1.41 ± 0.4	1.59 ± 0.4

* = *p* < 0.05 vs Control; ° = *p* < 0.05 vs F20%.

**Table 2:** Mitochondrial respirometry in fasted rats.

	Control	F 20%	F 20% + AP
**Malate/Glutamate**			
State 3, ng atoms O/min/mg prot	38 ± 11	45 ± 5	45 ± 9
State 4, ng atoms O/min/mg prot	8 ± 0.6	13 ± 1.3 *	7 ± 1.4 °
Respiratory control rate, S3/S4	5.4 ± 1.6	3.4 ± 0.4 *	6.3 ± 0.2 °
ADP/O_2_, nMol ADP/atoms O/min	2.8 ± 0.4	2.6 ± 0.3	3.6 ± 0.1 *°
CCCP, ng AtO/min/mg prot	56 ± 12	65 ± 11	38 ± 9 *°
**Succinate/Rotenone**			
State 3, ng atoms O/min/mg prot	62 ± 15	66 ± 6	64 ± 9
State 4, ng atoms O/min/mg prot	12 ± 3	16 ± 1	16 ± 5
Respiratory control rate, S3/S4	5.1 ± 1	4.3 ± 0.3	4.3 ± 0.9
ADP/O_2_, nMol ADP/atoms O/min	1.6 ± 0.2	1.4 ± 0.02	1.7 ± 0.2 °
CCCP, ng AtO/min/mg prot	126 ± 28	132 ± 18	124 ± 21

* = *p* < 0.05 vs Control; ° = *p* < 0.05 vs F20%.
